# 1,3-Dibenzyl­imidazolidine-2-thione

**DOI:** 10.1107/S1600536812032655

**Published:** 2012-07-21

**Authors:** Anna Mietlarek-Kropidłowska, Jaroslaw Chojnacki, Barbara Becker

**Affiliations:** a Department of Inorganic Chemistry, Chemical Faculty, Gdansk University of Technology, 11/12 G. Narutowicza Street, 80-233 Gdańsk, Poland

## Abstract

In the title compound, C_17_H_18_N_2_S, the imidazolidine ring adopts a twisted conformation. In the crystal, mol­ecules are linked by slipped π–π inter­actions between the benzene rings of neighbouring mol­ecules [centroid-to-centroid distance = 3.903 (2) Å].

## Related literature

For background information and the synthesis of related compounds, see: Savjani & Gajjar (2011 ▶); Wazeer *et al.* (2007 ▶); Zhivotova *et al.* (2006 ▶); Jayaram *et al.* (2008 ▶). For ring-puckering parameters, see: Cremer & Pople (1975 ▶).
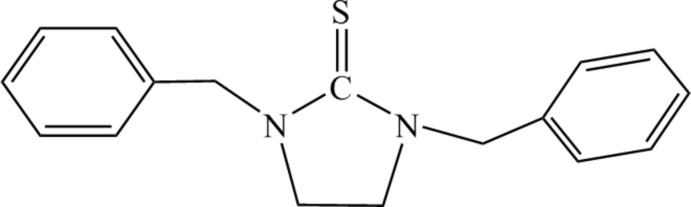


## Experimental

### 

#### Crystal data

C_17_H_18_N_2_S
*M* *_r_* = 282.39
Monoclinic, 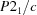

*a* = 14.8492 (8) Å
*b* = 10.2284 (5) Å
*c* = 10.1314 (6) Å
β = 107.131 (6)°
*V* = 1470.53 (14) Å^3^
*Z* = 4
Mo *K*α radiation
μ = 0.21 mm^−1^
*T* = 120 K
0.45 × 0.15 × 0.03 mm

#### Data collection

Oxford Xcalibur Sapphire2 diffractometer
Absorption correction: analytical (*CrysAlis PRO*; Oxford Diffraction, 2010 ▶) *T*_min_ = 0.938, *T*_max_ = 0.993
5840 measured reflections
2890 independent reflections
2148 reflections with *I* > 2σ(*I*)
*R*_int_ = 0.020

#### Refinement

*R*[*F* ^2^ > 2σ(*F* ^2^)] = 0.037
*wR*(*F* ^2^) = 0.092
*S* = 0.94
2890 reflections
181 parameters
H-atom parameters constrained
Δρ_max_ = 0.32 e Å^−3^
Δρ_min_ = −0.17 e Å^−3^

### 

Data collection: *CrysAlis PRO* (Oxford Diffraction, 2010 ▶); cell refinement: *CrysAlis PRO*; data reduction: *CrysAlis PRO*; program(s) used to solve structure: *SHELXS97* (Sheldrick, 2008 ▶); program(s) used to refine structure: *SHELXL97* (Sheldrick, 2008 ▶); molecular graphics: *ORTEP-3* (Farrugia, 1997 ▶); software used to prepare material for publication: *WinGX* (Farrugia, 1999 ▶).

## Supplementary Material

Crystal structure: contains datablock(s) global, I. DOI: 10.1107/S1600536812032655/lx2257sup1.cif

Structure factors: contains datablock(s) I. DOI: 10.1107/S1600536812032655/lx2257Isup2.hkl

Supplementary material file. DOI: 10.1107/S1600536812032655/lx2257Isup3.cml

Additional supplementary materials:  crystallographic information; 3D view; checkCIF report

## Figures and Tables

**Fig. 1.**
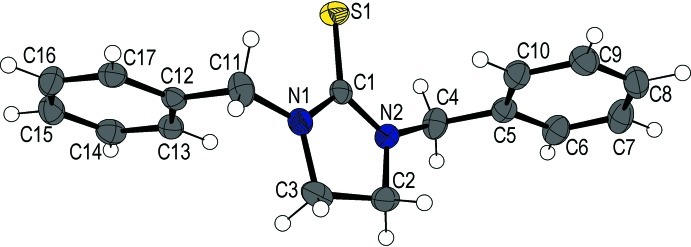
The molecular structure of the title compound with the atom numbering scheme. Displacement ellipsoids are drawn at the 50% probability level. H atoms are presented as small spheres of arbitrary radius.

**Fig. 2.**
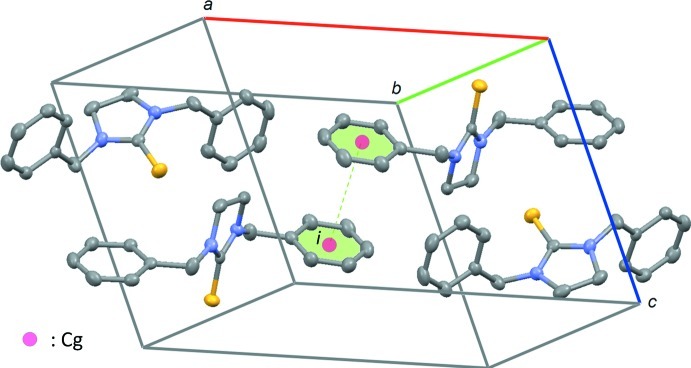
A view of the π–π interactions (dotted lines) in the crystal structure of the title compound. H atoms non-participating in hydrogen-bonding were omitted for clarity. Cg is the centroid of the C5–C10 benzene ring. [Symmetry code: (i) -x + 1, -y + 1, -z + 1.]

## supplementary crystallographic information

### Comment

2-Imidazolidinethione derivatives exhibit applications in diverse therapeutic areas such as antimicrobial activity (Wazeer *et al.*, 2007). Moreover, 2-imidazolidinethiones are also used as a chiral auxiliary and ligands for asymmetric catalysis (Savjani & Gajjar, 2011). Herein, we report the crystal structure of the title compound.

In the title molecule (Fig. 1), the imidazolidine ring has twisted (*T, i.e.* half-chair) conformation. In the crystal structure (Fig. 2), molecules are connected by slipped π–π interactions between the benzene rings of neighbouring molecules, with a Cg–Cg^i^ distance of 3.903 (2) Å and an interplanar distance of 3.595 (2) Å resulting in a slippage of 1.519 Å (Cg is the centroid of the C5–C10 benzene ring).

The volume 1470.53 (14) Å^3^ as well as the number of molecules in the elemental cell (*Z* = 4) of 1,3-dibenzylimidazolidine-2-thione match the values determined for closely related 1,3-dibenzyl-1*H*-imidazole-2(3*H*)-thione (Jayaram *et al.*, 2008). These molecules differ in their 5-membered ring being either aromatic or aliphatic. Nevertheless any closer comparison of the bond lengths and angles between these two compounds is difficult due to the lack of atomic coordinates for 1,3-dibenzyl-1*H*-imidazole-2(3*H*)-thione either in the above mentioned paper or in Cambridge Structural Database. The 5-membered imidazolidine ring in the present structure adopts the conformation which is most closely described as half-chair or twisted (*T*) on C2—C3. Parameter *Q_2_* (Cremer & Pople, 1975), which specifies the puckering amplitude and thus differentiate planar from non-planar systems, is significantly greater than zero 0.1565 (16) Å and φ_2_ parameter is 301.2 (6)° pointing to the mentioned *T* type of pucker.

### Experimental

The title compound was synthesized according to the procedure reported by Zhivotova *et al.* (2006). The reaction was carried out between N,N'-dibenzylethylenediamine and carbon disulfide in the presence of KOH (molar ratio 1:1:1) in methanol. The mixture was stirred 50 min, filtered and then left for crystallization at 278 K. After a week yellowish needle-like crystals were appeared. These were filtered off and dried. The melting point was determined to be 393 K.

### Refinement

All of the C-bonded hydrogen atoms were placed in the calculated positions (aromatic: *d*_CH_ = 0.95 Å, methylene: *d*_CH_ = 0.99 Å) and were treated as riding on their parent atoms with *U*_iso_(H) = 1.2*U*_eq_(C).

### Crystal data

**Table:**

C_17_H_18_N_2_S	*F*(000) = 600
*M**_r_* = 282.39	*D*_x_ = 1.276 Mg m^−^^3^
Monoclinic, *P*2_1_/*c*	Melting point: 393 K
Hall symbol: -P 2ybc	Mo *K*α radiation, λ = 0.71073 Å
*a* = 14.8492 (8) Å	Cell parameters from 3149 reflections
*b* = 10.2284 (5) Å	θ = 2.9–28.3°
*c* = 10.1314 (6) Å	µ = 0.21 mm^−^^1^
β = 107.131 (6)°	*T* = 120 K
*V* = 1470.53 (14) Å^3^	Needle, light yellow
*Z* = 4	0.45 × 0.15 × 0.03 mm

### Data collection

**Table:**

Oxford Xcalibur Sapphire2 diffractometer	2890 independent reflections
Graphite monochromator	2148 reflections with *I* > 2σ(*I*)
Detector resolution: 8.1883 pixels mm^-1^	*R*_int_ = 0.020
ω scans	θ_max_ = 26°, θ_min_ = 2.9°
Absorption correction: analytical (*CrysAlis PRO*; Oxford Diffraction, 2010)	*h* = −17→18
*T*_min_ = 0.938, *T*_max_ = 0.993	*k* = −11→12
5840 measured reflections	*l* = −11→12

### Refinement

**Table:**

Refinement on *F*^2^	Primary atom site location: structure-invariant direct methods
Least-squares matrix: full	Secondary atom site location: difference Fourier map
*R*[*F*^2^ > 2σ(*F*^2^)] = 0.037	Hydrogen site location: inferred from neighbouring sites
*wR*(*F*^2^) = 0.092	H-atom parameters constrained
*S* = 0.94	*w* = 1/[σ^2^(*F*_o_^2^) + (0.0605*P*)^2^] where *P* = (*F*_o_^2^ + 2*F*_c_^2^)/3
2890 reflections	(Δ/σ)_max_ = 0.001
181 parameters	Δρ_max_ = 0.32 e Å^−^^3^
0 restraints	Δρ_min_ = −0.17 e Å^−^^3^

### Special details

**Table:**

Geometry. All s.u.'s (except the s.u. in the dihedral angle between two l.s. planes) are estimated using the full covariance matrix. The cell s.u.'s are taken into account individually in the estimation of s.u.'s in distances, angles and torsion angles; correlations between s.u.'s in cell parameters are only used when they are defined by crystal symmetry. An approximate (isotropic) treatment of cell s.u.'s is used for estimating s.u.'s involving l.s. planes.
Refinement. Refinement of *F*^2^ against ALL reflections. The weighted *R*-factor *wR* and goodness of fit *S* are based on *F*^2^, conventional *R*-factors *R* are based on *F*, with *F* set to zero for negative *F*^2^. The threshold expression of *F*^2^ > 2σ(*F*^2^) is used only for calculating *R*-factors(gt) *etc*. and is not relevant to the choice of reflections for refinement. *R*-factors based on *F*^2^ are statistically about twice as large as those based on *F*, and *R*-factors based on ALL data will be even larger.

### Fractional atomic coordinates and isotropic or equivalent isotropic displacement parameters (Å^2^)

**Table:**

	*x*	*y*	*z*	*U*_iso_*/*U*_eq_	
N1	0.06996 (8)	0.45939 (12)	0.28050 (13)	0.0281 (3)	
N2	0.20826 (8)	0.36922 (12)	0.37091 (13)	0.0268 (3)	
S1	0.11232 (3)	0.27228 (4)	0.12140 (4)	0.03308 (14)	
C1	0.13043 (10)	0.36852 (14)	0.26067 (15)	0.0233 (3)	
C2	0.20697 (11)	0.47669 (16)	0.46506 (17)	0.0334 (4)	
H2A	0.2501	0.5478	0.4565	0.04*	
H2B	0.2246	0.4464	0.5621	0.04*	
C3	0.10445 (11)	0.52096 (16)	0.41609 (17)	0.0335 (4)	
H3A	0.0692	0.4899	0.4791	0.04*	
H3B	0.0997	0.6174	0.4088	0.04*	
C4	0.28837 (10)	0.28334 (16)	0.38936 (18)	0.0329 (4)	
H4A	0.2685	0.2064	0.3284	0.039*	
H4B	0.3081	0.2515	0.4859	0.039*	
C5	0.37268 (10)	0.34615 (14)	0.35870 (16)	0.0270 (3)	
C6	0.46349 (11)	0.30853 (16)	0.43384 (18)	0.0338 (4)	
H6	0.4721	0.246	0.5059	0.041*	
C7	0.54126 (11)	0.36151 (16)	0.40445 (19)	0.0388 (4)	
H7	0.6028	0.3343	0.4555	0.047*	
C8	0.52966 (11)	0.45327 (17)	0.30165 (19)	0.0388 (4)	
H8	0.5832	0.49	0.2824	0.047*	
C9	0.43962 (12)	0.49221 (17)	0.22599 (18)	0.0365 (4)	
H9	0.4314	0.5558	0.1551	0.044*	
C10	0.36144 (11)	0.43758 (16)	0.25455 (16)	0.0328 (4)	
H10	0.2999	0.4634	0.202	0.039*	
C11	−0.02190 (10)	0.48742 (16)	0.18592 (18)	0.0341 (4)	
H11A	−0.0199	0.4689	0.0909	0.041*	
H11B	−0.0345	0.582	0.1912	0.041*	
C12	−0.10361 (10)	0.41236 (14)	0.21001 (15)	0.0234 (3)	
C13	−0.09255 (10)	0.31025 (14)	0.30325 (15)	0.0259 (3)	
H13	−0.0311	0.2852	0.3572	0.031*	
C14	−0.17071 (11)	0.24423 (15)	0.31840 (17)	0.0317 (4)	
H14	−0.1623	0.1741	0.3824	0.038*	
C15	−0.26067 (11)	0.27991 (17)	0.24095 (18)	0.0360 (4)	
H15	−0.3141	0.2351	0.2517	0.043*	
C17	−0.27181 (10)	0.38191 (17)	0.14751 (17)	0.0358 (4)	
H17	−0.3333	0.4069	0.0939	0.043*	
C18	−0.19466 (10)	0.44741 (15)	0.13154 (16)	0.0285 (4)	
H18	−0.2034	0.5169	0.0668	0.034*	

### Atomic displacement parameters (Å^2^)

**Table:**

	*U*^11^	*U*^22^	*U*^33^	*U*^12^	*U*^13^	*U*^23^
N1	0.0252 (6)	0.0280 (7)	0.0329 (8)	−0.0007 (5)	0.0113 (5)	−0.0046 (6)
N2	0.0244 (6)	0.0293 (7)	0.0257 (7)	−0.0010 (5)	0.0059 (5)	−0.0015 (6)
S1	0.0371 (2)	0.0336 (2)	0.0283 (2)	−0.00352 (17)	0.00927 (17)	−0.00815 (18)
C1	0.0240 (7)	0.0229 (7)	0.0252 (8)	−0.0053 (6)	0.0109 (6)	0.0004 (6)
C2	0.0391 (9)	0.0360 (9)	0.0276 (9)	−0.0154 (7)	0.0139 (7)	−0.0060 (7)
C3	0.0452 (10)	0.0290 (8)	0.0328 (9)	−0.0040 (7)	0.0214 (8)	−0.0040 (7)
C4	0.0266 (8)	0.0317 (9)	0.0375 (9)	0.0010 (7)	0.0050 (7)	0.0085 (8)
C5	0.0265 (8)	0.0245 (8)	0.0288 (9)	0.0006 (6)	0.0064 (6)	−0.0026 (6)
C6	0.0293 (8)	0.0303 (9)	0.0384 (10)	0.0034 (7)	0.0044 (7)	0.0008 (7)
C7	0.0237 (8)	0.0355 (10)	0.0542 (12)	0.0050 (7)	0.0066 (7)	−0.0051 (9)
C8	0.0305 (9)	0.0398 (10)	0.0509 (11)	−0.0061 (7)	0.0196 (8)	−0.0116 (8)
C9	0.0388 (9)	0.0378 (10)	0.0348 (10)	−0.0027 (7)	0.0138 (7)	0.0011 (8)
C10	0.0270 (8)	0.0373 (9)	0.0321 (9)	0.0003 (7)	0.0057 (7)	0.0012 (7)
C11	0.0282 (8)	0.0341 (9)	0.0418 (10)	0.0062 (7)	0.0133 (7)	0.0128 (8)
C12	0.0254 (7)	0.0228 (7)	0.0236 (8)	0.0029 (6)	0.0095 (6)	−0.0034 (6)
C13	0.0267 (8)	0.0257 (8)	0.0252 (8)	0.0032 (6)	0.0076 (6)	0.0006 (6)
C14	0.0406 (9)	0.0279 (8)	0.0294 (9)	−0.0035 (7)	0.0145 (7)	−0.0003 (7)
C15	0.0310 (8)	0.0375 (9)	0.0433 (10)	−0.0097 (7)	0.0167 (7)	−0.0102 (8)
C17	0.0242 (8)	0.0425 (10)	0.0371 (10)	0.0022 (7)	0.0036 (7)	−0.0073 (8)
C18	0.0316 (8)	0.0291 (8)	0.0241 (8)	0.0052 (7)	0.0072 (6)	−0.0008 (7)

### Geometric parameters (Å, º)

**Table:**

N1—C1	1.3479 (18)	C7—H7	0.95
N1—C11	1.446 (2)	C8—C9	1.389 (2)
N1—C3	1.460 (2)	C8—H8	0.95
N2—C1	1.3501 (19)	C9—C10	1.393 (2)
N2—C4	1.4459 (18)	C9—H9	0.95
N2—C2	1.4591 (19)	C10—H10	0.95
S1—C1	1.6759 (15)	C11—C12	1.515 (2)
C2—C3	1.524 (2)	C11—H11A	0.99
C2—H2A	0.99	C11—H11B	0.99
C2—H2B	0.99	C12—C13	1.385 (2)
C3—H3A	0.99	C12—C18	1.398 (2)
C3—H3B	0.99	C13—C14	1.390 (2)
C4—C5	1.518 (2)	C13—H13	0.95
C4—H4A	0.99	C14—C15	1.384 (2)
C4—H4B	0.99	C14—H14	0.95
C5—C10	1.383 (2)	C15—C17	1.385 (2)
C5—C6	1.393 (2)	C15—H15	0.95
C6—C7	1.385 (2)	C17—C18	1.377 (2)
C6—H6	0.95	C17—H17	0.95
C7—C8	1.375 (3)	C18—H18	0.95
			
C1—N1—C11	125.29 (13)	C6—C7—H7	119.9
C1—N1—C3	111.91 (12)	C7—C8—C9	119.96 (15)
C11—N1—C3	122.62 (13)	C7—C8—H8	120
C1—N2—C4	125.09 (13)	C9—C8—H8	120
C1—N2—C2	111.84 (12)	C8—C9—C10	119.72 (16)
C4—N2—C2	122.81 (12)	C8—C9—H9	120.1
N1—C1—N2	108.57 (13)	C10—C9—H9	120.1
N1—C1—S1	125.58 (12)	C5—C10—C9	120.59 (14)
N2—C1—S1	125.84 (11)	C5—C10—H10	119.7
N2—C2—C3	102.42 (12)	C9—C10—H10	119.7
N2—C2—H2A	111.3	N1—C11—C12	115.89 (13)
C3—C2—H2A	111.3	N1—C11—H11A	108.3
N2—C2—H2B	111.3	C12—C11—H11A	108.3
C3—C2—H2B	111.3	N1—C11—H11B	108.3
H2A—C2—H2B	109.2	C12—C11—H11B	108.3
N1—C3—C2	102.60 (12)	H11A—C11—H11B	107.4
N1—C3—H3A	111.2	C13—C12—C18	118.78 (13)
C2—C3—H3A	111.2	C13—C12—C11	123.52 (13)
N1—C3—H3B	111.2	C18—C12—C11	117.68 (13)
C2—C3—H3B	111.2	C12—C13—C14	120.44 (14)
H3A—C3—H3B	109.2	C12—C13—H13	119.8
N2—C4—C5	114.39 (12)	C14—C13—H13	119.8
N2—C4—H4A	108.7	C15—C14—C13	120.48 (15)
C5—C4—H4A	108.7	C15—C14—H14	119.8
N2—C4—H4B	108.7	C13—C14—H14	119.8
C5—C4—H4B	108.7	C14—C15—C17	119.14 (14)
H4A—C4—H4B	107.6	C14—C15—H15	120.4
C10—C5—C6	118.92 (14)	C17—C15—H15	120.4
C10—C5—C4	121.38 (13)	C18—C17—C15	120.70 (14)
C6—C5—C4	119.67 (14)	C18—C17—H17	119.7
C7—C6—C5	120.57 (16)	C15—C17—H17	119.7
C7—C6—H6	119.7	C17—C18—C12	120.46 (14)
C5—C6—H6	119.7	C17—C18—H18	119.8
C8—C7—C6	120.24 (15)	C12—C18—H18	119.8
C8—C7—H7	119.9		
			
C11—N1—C1—N2	−179.34 (13)	C5—C6—C7—C8	0.8 (3)
C3—N1—C1—N2	−4.12 (17)	C6—C7—C8—C9	−0.6 (3)
C11—N1—C1—S1	1.4 (2)	C7—C8—C9—C10	−0.2 (3)
C3—N1—C1—S1	176.58 (11)	C6—C5—C10—C9	−0.6 (2)
C4—N2—C1—N1	178.56 (12)	C4—C5—C10—C9	−178.66 (15)
C2—N2—C1—N1	−7.22 (16)	C8—C9—C10—C5	0.8 (2)
C4—N2—C1—S1	−2.2 (2)	C1—N1—C11—C12	91.76 (18)
C2—N2—C1—S1	172.08 (10)	C3—N1—C11—C12	−82.97 (18)
C1—N2—C2—C3	14.63 (16)	N1—C11—C12—C13	−8.5 (2)
C4—N2—C2—C3	−170.99 (13)	N1—C11—C12—C18	172.64 (13)
C1—N1—C3—C2	12.79 (16)	C18—C12—C13—C14	−0.1 (2)
C11—N1—C3—C2	−171.84 (13)	C11—C12—C13—C14	−178.92 (14)
N2—C2—C3—N1	−15.51 (14)	C12—C13—C14—C15	−0.3 (2)
C1—N2—C4—C5	101.77 (17)	C13—C14—C15—C17	0.3 (2)
C2—N2—C4—C5	−71.85 (19)	C14—C15—C17—C18	−0.1 (2)
N2—C4—C5—C10	−35.3 (2)	C15—C17—C18—C12	−0.3 (2)
N2—C4—C5—C6	146.64 (14)	C13—C12—C18—C17	0.4 (2)
C10—C5—C6—C7	−0.2 (2)	C11—C12—C18—C17	179.26 (15)
C4—C5—C6—C7	177.88 (15)		
